# Biodegradable Poly (lactic acid)/Poly (ethylene glycol) Reinforced Multi-Walled Carbon Nanotube Nanocomposite Fabrication, Characterization, Properties, and Applications

**DOI:** 10.3390/polym12020427

**Published:** 2020-02-12

**Authors:** Ahmad Fahad Ahmad, Sidek Ab Aziz, Suzan Jabbar Obaiys, Mohd Hafiz Mohd Zaid, Khamirul Amin Matori, Kanagesan Samikannu, Umar Sa’as Aliyu

**Affiliations:** 1 Department of Physics, Faculty of Science, Universiti Putra Malaysia, Serdang 43400, Malaysia; mhmzaid@upm.edu.my (M.H.M.Z.); khamirul@upm.edu.my (K.A.M.); 2 School of Mathematical & Computer Sciences, Heriot-Watt University Malaysia, Putrajaya 62200, Malaysia; s.obaiys@hw.ac.uk; 3 Low Dimensional Materials Research Centre, Department of Physics, Faculty of Science, University of Malaya, Kuala Lumpur 50603, Malaysia; kanagu1980@um.edu.my; 4 Department of Physics, Faculty of Science, Federal University Lafia, Nasarawa State, 0146 Lafia, Nigeria; usaltilde@yahoo.com

**Keywords:** poly (lactic acid), multi-walled carbon nanotubes, nanocomposites, poly (ethylene glycol)

## Abstract

This paper presents the electromagnetic interference properties of multi-walled carbon nanotubes (MWCNTs) as a novel nano-reinforcement filler in poly (lactic acid) (PLA)/poly (ethylene glycol) (PEG) polymer matrix that was prepared via melt blending mode. Plasticization of PLA was first carried out by PEG, which overcomes its brittleness problem, in order to enhance its flexibility. A waveguide adapter technique was used to measure the dielectric properties $\varepsilon_{r}$, and S-parameters reflection (S_11_) and transmission (S_21_) coefficients. The dielectric properties, microwave attenuation performances, and electromagnetic interference shielding effectiveness (EMISE) for all the material under test have been calculated over the full X-Band (8–12 GHz) due to its importance for military and commercial applications. The prepared samples were studied while using X-ray diffraction (XRD), field emission scanning electron microscopy (FE-SEM), Fourier transforms infrared spectroscopy (FTIR), mechanical properties measurements, as well as thermogravimetric analysis (TGA). The results showed that the dielectric properties increased with increased multi-walled carbon nanotubes (MWCNTs) filler, as well as the shielding effectiveness of the MWCNT/PLA/PEG nanocomposites increased with the increasing of MWCNTs. The highest SE total value was found to be 42.07 dB at 12 GHz for 4 wt.% filler content. It is also observed that the attenuation values of the nanocomposites increased with an increase in MWCNTs loading, as well as the power loss values for all of the samples increased with the increase in MWCNTs loading, except the amount of the transmitted wave through the nanocomposites.

## 1. Introduction

Electromagnetic interference (EMI) shielding is considered to be very important in today’s electronic components and devices [1], where these electronic devices are radiated and they are affected by electromagnetic interference (EMI). It is essential to protect such devices from being irradiated by EMI, so as to maintain their integrity and functionality. Additionally, it is required to control the level of their EMI emission, for product acceptance by complying with the standards of electromagnetic compatibility that agencies of government impose [2]. Metal cabinets, conductive coatings, conductive polymer composites (CPCs), and foil laminates are the means of EMI shielding. There has been an extensive investigation regarding the use of carbon nanotubes in various matrices as reinforcements. In polymer composites, the commonly used conducting fillers are carbon black, with the major disadvantage being the high amount of carbon black that is required up to 30–40% to achieve desired conductivity levels, which results in polymers’ mechanical properties deterioration [3,4].

In recent years, a significant increase in the utilization of conductive material of carbon nanotubes (CNTs) for plastics in the sectors of automotive, electronics, and aerospace has been noted. In addition, that, they show potential for utilization as materials for EMI shielding due to their better processing advantages, resistance to corrosion, lightweight, and flexibility when compared with the conventional metal composites [5]. Carbon nanotubes (CNTs) present excellent electrical, mechanical, thermal, and structural properties, which result from the combination of various CNTs quantities and biodegradable polymers for second-phase reinforcement, which attracts great interest from both industrial and academic settings [6]. Good CNTs dispersion in the matrix of a polymer is of great importance when it comes to high-performance polymer nanocomposites. Not only is the interfacial adhesion between the matrix and CNTs improved by the homogeneous dispersion, but it also prevents CNTs aggregation, which negatively impacts the overall mechanical properties of the composites and causes heterogeneity [7].

Biopolymers have received great attention in both the industry and in academia due to increased concern toward the environmental impact of plastic waste and the saving of limited fossil energy. Poly (lactic acid) (PLA), especially in applications, like the packaging, is one of the frequently used biodegradable polymers. This is mostly due to their high modulus of elasticity, high strength, processability, good optical transparency, and biocompatibility. However, despite the mentioned qualities, PLA also has some kinds of drawbacks, which include poor toughness and inherent brittleness, impeding its wide applications. A lot of effects have been made toward the improvement of the PLA properties in order for the material to compete with low cost and commodity flexibility. These attempts include blending with inorganic nano-fillers, modifying PLA with plasticizers, or blending PLA with other polymers [8]. Poly (ethylene glycol) (PEG) is the potential plasticizer for PLA and it can either be in the form of a solid or liquid polymer. It has been observed that PEG has shown great promise as PLA plasticizing agents by giving a large elongation increase at break [9]. Additionally, studies regarding their tensile properties revealed that PEG-200 addition led to an elongation increase at the break, but a decrease in both tensile modulus and tensile strength. However, there was no stability in PLA/PEG blend and there was loss in terms of their attractive properties at ambient temperatures over time, due to phase separation at such temperatures leading to PEG-rich and PLA-rich phases’ formation [10]. 

There are several reports on the study of CNTs-based polymer composites’ EMI shielding properties. Some of the reports showed that the conducting composites’ formation ability at low CNTs loading, which results from low thresholds of percolation, is the major advantage of CNTs use [11]. For that reason, studies in so many quarters on multi-walled carbon nanotubes (MWCNTs) incorporated nanocomposites are ongoing as it provides good conductive properties. This is in addition to the typically derived advantages from the high ratio of surface to volume of the reinforcing phase. However, it is common knowledge that nanocomposites of this nature present some serious problems that have to do with high CNTs agglomeration trend [12].

Yang et al. [13] reported a study on the CNTs-PS foam composites’ EMI shielding applications and the result showed a value of around 20 dB at 7 wt.% loading. The composites appeared to be more reflective of electromagnetic radiation than absorptive. Bryant et al. [14] studied the effects of different carbon nanofiber and CNT contents within the PS matrix on the EMI shielding effectiveness (SE). The obtained result showed 20.3 dB SE value for 1 mm sample thickness with 1 wt.% CNTs addition in 10 wt.% carbon nanofiber polystyrene composites. Huang et al. [15] prepared composites of SWCNTs–epoxy with different walls integrities and aspect ratios while using short, long, and annealed Single-walled carbon nanotubes (SWCNTs). In the X-band range, 20–30 dB EMI SE and very low percolation volumes were recorded for 15 wt.% SWCNTs loading. Liu et al. reported up to about 17 dB, EMI was reported [16] in 8.2–12.4 GHz band with 20 wt.% SWCNTs loading for PU/SWCNTs composites. It is quite evident from the reports that the values for EMI shielding vary mostly between 20 to 30 dB for MSWCNTs or SWCNTs polymer nanocomposites in the X-band frequency region. However, for frequencies other than the X-band, reports of higher values were presented [17]. 

The present study has three main goals: (a) prepare polymer nanocomposites from a biodegradable polymer in the form of poly(lactic acid) (PLA) that was mixed with poly (ethylene e glycol) (PEG) used as a plasticizer to soften and reduce the brittleness of PLA to the possibility of manufacturing samples easily, and then fill with the MWCNTs nanoparticle, (b) study the physical, mechanical, and structural properties of the composites while using Fourier transform infrared spectroscopy (FT-IR), X-ray diffraction (XRD), as well as Field-emission scanning electron microscopy (FE-SEM) (c) Study the EMI shielding efficiency, dielectric, and the conductivity properties of the nanocomposite at X-band frequency while using a rectangular waveguide technique. This technique enables the accurate extraction of the material’s properties from the measured S-parameters. The effects of the MWCNTs loading on the overall dielectric properties and the EMI shielding mechanism were investigated.

## 2. Materials and Methods

### 2.1. Materials

**Figure 1:**
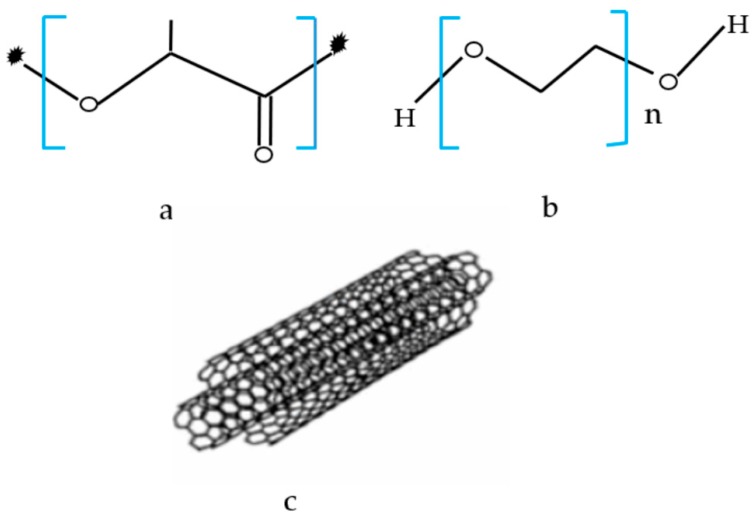
Chemical formula of (**a**) pure poly (lactic acid) (PLA), (**b**) pure poly (ethylene glycol) (PEG), and (**c**) multi-walled carbon nanotubes (MWCNTs) structure.

The materials that were used in this work included: poly(lactic acid) (PLA) pellets with a density of 1.24 g/cm^3^ (Grade 4060D) from Nature Work LLC (Minnetonka, MN, USA); low molecular weight poly (ethylene glycol) (PEG) (Mn = 200 g/mol) was purchased from Sigma-Aldrich (St. Louis, MO, USA); PLA/PEG blend with a melting temperature between 160–180 °C was used as the base polymer matrix; and, MWCNT was purchased from Sigma-Aldrich (St. Louis, MO, USA) having an average diameter of 9.5 nm. Figure 1 illustrates the chemical structures of polymers and MWCNTs used in this study in.

### 2.2. Preparation of MWCNT/PLA/PEG Nanocomposites

**Figure 2:**
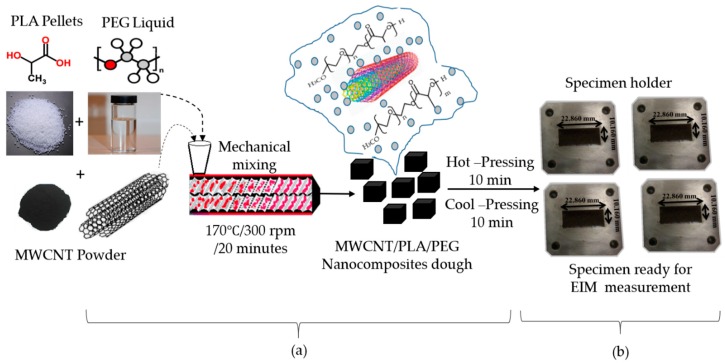
Schematic representation illustrating (**a**) the preparation of MWCNT/PLA/PEG nanocomposites, Chemical structure of materials as- preparation, and (**b**) MWCNT/PLA/PEG rectangular specimens fit inside the sample holder for electromagnetic interference (EMI) measurement.

In this study, the MWCNT/PLA/PEG nanocomposites were prepared while using melt blending technique, using Brabender internal mixer (GmbH & Co. KG, Duisburg, Germany), at 50 rpm of the rotor speed, at 170 °C for 20 min. of time mixing. The obtained blends were then molded into sheets of 1 mm in thickness by hot pressing at 170 °C for 5 min. with the pressure of 110 kg/cm^2^, followed by cooling to room temperature. Subsequently, the sheets were used for further characterization. The dispersion of MWCNTs nanoparticles as a filler was within the ratio 9:1 for PLA/PEG polymer, as illustrated in Figure 2a. The MWCNTs/PLA/PEG nanocomposites with different content of MWCNTs were fabricated into rectangular shapes molds with a dimension of 22.86 mm × 10.16 mm × 3 mm to study the dielectric properties, as shown in Figure 2b. Table 1 shows the percentage of MWCNTs nanoparticles that mixed together with the PLA/PEG polymer matrix.

### 2.3. Measurement Setup

**Figure 3:**
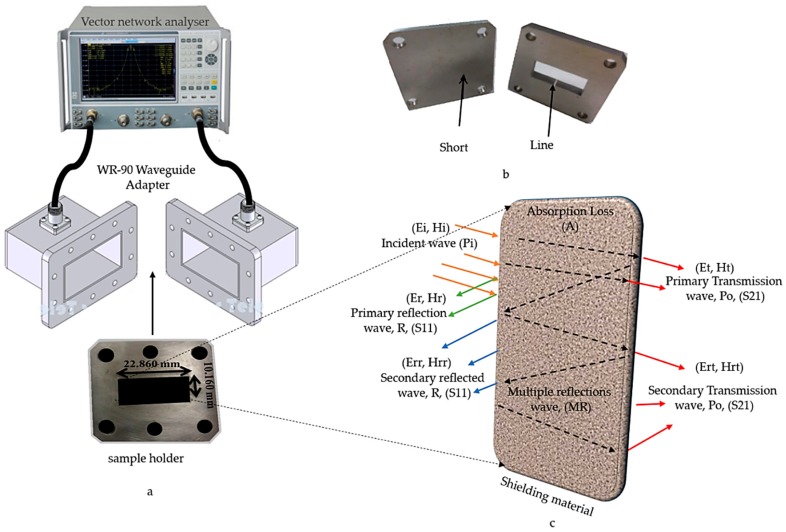
(**a**) Electromagnetic properties measurement setup, (**b**) Thru-Reflect-Load (TRL) calibration Line and short for rectangular waveguide, and (**c**) Schematic showing the mechanism of the passage of the wave inside the material under test.

The EMI measurement for the nanocomposites was divided into three parts: the first part starts with the scattering parameters correspond to the reflected (S_11_) and transmitted (S_21_) powers; the second part of the measurement was shielding effectiveness (SE) calculated based on the results of the first part and they are computed using commercial measurement software, the Agilent N5230A PNA-L network analyzer system and Agilent 85701B software package [18]; and, the third part starts with the dielectric (real and imaginary parts) properties computed while using commercial measurement software (Agilent 85071E) software. Figure 3 schematically illustrates the measurement steps, where the sample holder is placed between the WR-90 waveguide adapters that are connected to the vector network analyzer via a coaxial cable, as shown in Figure 3a. The correction, as well as handling of all the measurement errors at vector network analyzer, are calibrated before the measurement process by the Thru-Reflect-Load (TRL), which is illustrated in Figure 3b. Figure 3c describes the mechanism of shielding (EMI SE) when the wave passes within the material. The nanocomposite samples were tested at 201 data points and the data were taken within a frequency range of 8 to 12 GHz, because the shielding effectiveness in this range of frequencies is important in military, medical, and commercial applications [19].

### 2.4. Nanocomposites Characterization

#### 2.4.1. Tensile Properties Measurement

The test for tensile properties was carried out while using Instron 4302 series IX (Buckinghamshire, UK). With the help of the ASTM D638 (type V) standard, the samples were cut into a dumbbell shape. 1.0 kN load was applied at room temperature and at a constant speed of 10 mm/min. of the crosshead. At break, the evaluation of the tensile modulus, tensile strength, and elongation was carried out from the stress-strain data. Five tested replicates of each sample were included in order to obtain reliable standard and mean deviations.

#### 2.4.2. X-ray Diffraction (XRD)

The measurement of X-ray diffraction performed by using a Bruker diffractometer 163 ((Yuseong, Daejeon, Korea), being operated at 30 mA and 30 kV with CuKα radiation (λ = 1.542 Å). The data were recorded in 2θ range of 20°–80° at the scan rate of 2°/min.

#### 2.4.3. Fourier Transform Infrared (FT-IR)

The spectra of FT-IR were recorded while using the Perkin Wlmer BX (Waltham, MA, USA) Version of a FT-IR spectrometer that was equipped with a universal attenuated total reflectance. The spectra recorded were between the wavenumber range of 400 and 4000 cm^−1^.

#### 2.4.4. Thermogravimetric Analysis (TGA and DTG) Properties

Thermogravimetric analysis (TGA) is a technique of thermal analysis that has to do with measurements of the sample’s mass changes in a controlled atmosphere with increasing temperature. The derivative curve that indicates the thermal degradation’s starting point is called derivative thermogravimetric (DTG). The results’ recording (for isothermal analysis) are made as weight loss–time or mass loss-temperature (for constant speed heating analysis). TGA analysis was performed while using a Perkin Elmer Pyris 7 TGA analyzer (1600LF, Shanghai Mettler Toledo Co. Ltd, China) with a 20 to 600 °C scan range, at 10 °C/min. constant heating rate, and continuous flow of nitrogen. The onset temperature (Tonset) and the maximum weight loss temperature (Tmax) were the temperatures of thermal degradation recorded.

#### 2.4.5. Field Emission Scanning Electron Microscopy (FE-SEM)

Field-emission scanning electron microscope (FE-SEM, JSM-6400 (Tokyo, Japan)) at an accelerating voltage of 30 kV was used to study the morphology, structure, and examine the interfacial adhesion between the matrix filler and polymer. The coating of the fractured surfaces was carried out while using a thin layer of gold prior to image analysis and observation.

## 3. Results and Discussion

### 3.1. X-ray Diffraction Analysis

**Figure 4:**
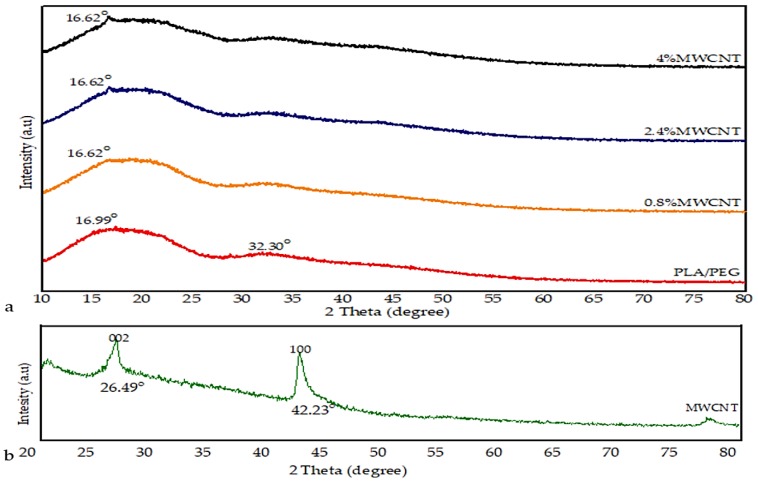
X-ray diffraction (XRD) pattern of (**a**) PLA/PEG, and MWCNT/PLA/PEG nanocomposites with different MWCNTs concentrations, and (**b**) MWCNTs powder.

The XRD patterns of the PLA/PEG mixture, MWCNTs powder, and MWCNT/PLA/PEG nanocomposites were evaluated and are presented in Figure 4. Figure 4a illustrates the spectrum of PLA/PEG, which was characterized by an attenuated broad peak at 16.99° and another peak centered at the 2θ value of 32.30° reflected that less sharpness corresponded to the typical spectrum of the PLA polymer characterized by broadband centered at the 2θ value of 16.99° with a relevant background [8]. The absence of a crystalline peak in PLA/PEG indicates amorphous nature due to the fact that PEG does not interfere with the crystalline state of the polyester [20].

In Figure 4b, it can be observed that the pattern of pure MWCNTs exhibited the typical peaks centered at the 2θ value of 26.49°, which corresponds to the (0 0 2) planes, the other peak centered around 42.23° is for (1 0 0) planes, corresponding to the graphite reflections (Joint Committee for Powder Diffraction Studies (JCPDS) No. 01-0646) [21]. On the other hand, XRD is used in the determination of the effect of the addition of MWCNTs nanoparticle to amorphous PLA/PEG materials. It was observed from the XRD result that the incorporation of the MWCNTs led to a gradual emergence of a new peak that increases in severity with and increase the MWCNTs loading. This implies that bonding interactions between PLA/PEG and MWCNTs may increase gradually with filler loading. Where the intensity of the diffraction peak becomes stronger and gradually increases with increased MWCNTs loading (0.8%, 2.4%, and 4%). The XRD results demonstrated that the amorphous structure of PLA/PEG mixture might be slightly changed to semi-crystalline with the incorporation of MWCNTs.

### 3.2. FE-SEM Observation

**Figure 5:**
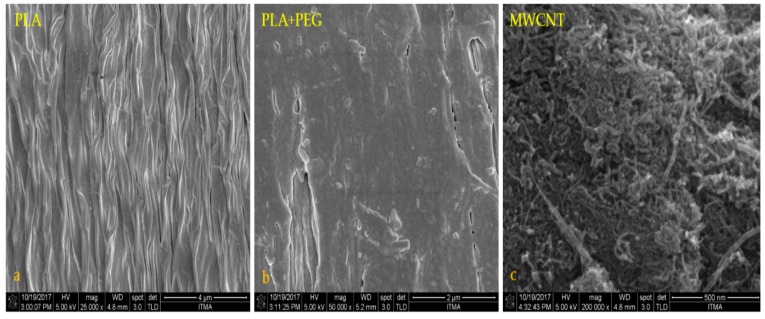

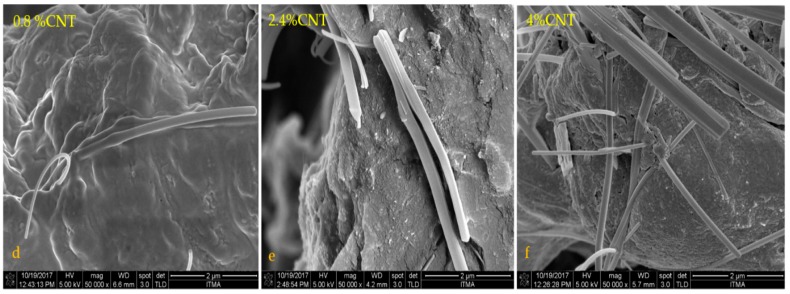
Field-emission scanning electron microscopy (FE-SEM) micrograph of (**a**) neat PLA, (**b**) PLA/PEG blend, (**c**) MWCNT powder, and the images of composite films with different MWCNT content (**d**) 0.8 wt.% MWCNT, (**e**) 2.4 wt.% MWCNT, and (**f**) 4 wt.% MWCNTs.

The morphology of the specimens from the impact tests was studied by field emission scanning electron microscopy (FE-SEM). Figure 5a–f shows FE-SEM images of the neat PLA slab, PLA/PEG blend, MWCNTs powder, and MWCNT/PLA/PEG nanocomposites at different percentages of MWCNTs filler. Figure 5a,b shows the FE-SEM micrograph of the PLA and PLA/PEG matrix, where the neat material shows rather brittle fracture surfaces with little plastic deformation; a few long threads of a deformed material are discernible on the fracture surfaces of these materials. The FE-SEM images reveal mats of fine nanotube that form a random, dense, and interconnected network. In Figure 5c, it can be observed that the MWCNTs are curvy and tangled with each other and MWCNTs nanoparticles have a high tendency to form bundles due to strong Van der Waals interactions [22,23]. The bundles of MWCNTs inside the PLA/PEG matrix also help to enhance the thermal stability of MWCNT/PLA/PEG nanocomposites. The dispersion of 0.8 wt.% and 2.4 wt.% content of MWCNTs in PLA/PEG matrix is better than 4 wt.% of MWCNTs, as shown in Figure 5d–f. The agglomeration of MWCNTs will block the wave transmission through the nanocomposites. This shows that the dispersion of MWCNTs in the matrix plays an important role in controlling nanocomposite properties. Furthermore, the proper dispersion of MWCNTs likely directly reflects the bonding interactions between polymer molecules and MWCNTs fillers [24].

### 3.3. Fourier Transform Infrared Spectroscopy (FT-IR) Analysis

**Figure 6:**
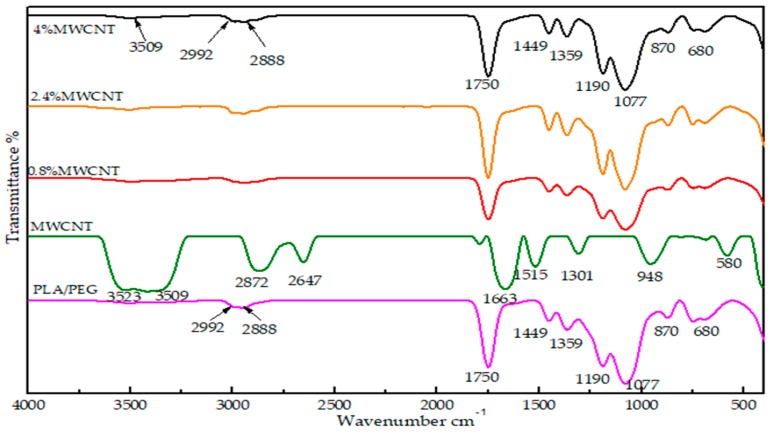
Fourier Transform Infrared (FT-IR) spectra of PLA/PEG blend, MWCNTs powder, and MWCNT/PLA/PEG nanocomposites with different MWCNTs loading (0.8 wt.%, 2.4 wt.%, and 4 wt.%).

FTIR was used to investigate the functional groups and bonding particles in MWCNTs powder, PLA/PEG mixture, and component in nanocomposites films at different MWCNTs loading (0.8 wt.% 2.4 wt.% and 4 wt.%). A number of interesting peaks were observed, and Figure 6 presents a representative spectrum. The spectra show intensive bands near 3385 cm^−1^ corresponding to the stretching vibrations of isolated surface OH substituents and/or OH of carboxyl groups and of adsorbed water. The IR band at 2992–2888 cm^−1^ corresponds to the symmetric stretching of C-H bonds in carbonaceous material. The shifts in characteristic wavenumbers to lower wavenumbers indicate the presence of strong hydrogen bonds between the OH groups. The bands in the 1794–1514 cm^−1^ range can be assigned to the carbonyl group of C=O bond in different environments, whereas the bands in the range of 1302–584 cm^−1^ confirmed the presence of CO bonds coming from various chemical surroundings. The peaks at 868 and 755 are related to the amorphous and crystalline regions [25]. It should be noted that the significant characteristic peaks of PLA/PEG were still dominant upon the addition of MWCNTs. The characteristic peaks that are responsible for –CH stretching, –C=O stretching, C–H bending, as well as –C–O stretching were clearly observed over the spectra for all the nanocomposites and no new peaks were formed with the increase in MWCNTs phase. This is expected, due to MWCNTs not presenting strong functional groups available to form a strong interface with a polymer matrix. Therefore, any property change of the nanocomposites is the result of the physical interaction between the MWCNTs and the PLA/PGE matrix, as well as can be ascribed to the charge transfer interactions between the conjugated surfaces of MWCNTs and the particles of PLA/PEG.

### 3.4. Thermogravimetric Analysis

**Figure 7:**
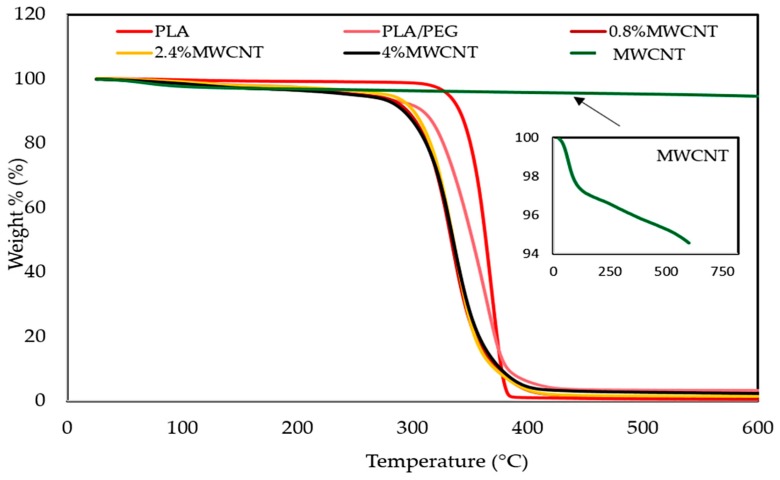
Thermogravimetric (TGA) curves of neat PLA, PLA/PEG mixture, MWCNTs powder, and nanocomposites with different MWCNTs loading (0.8 wt.%, 2.4 wt.% and 4 wt.%).

**Figure 8:**
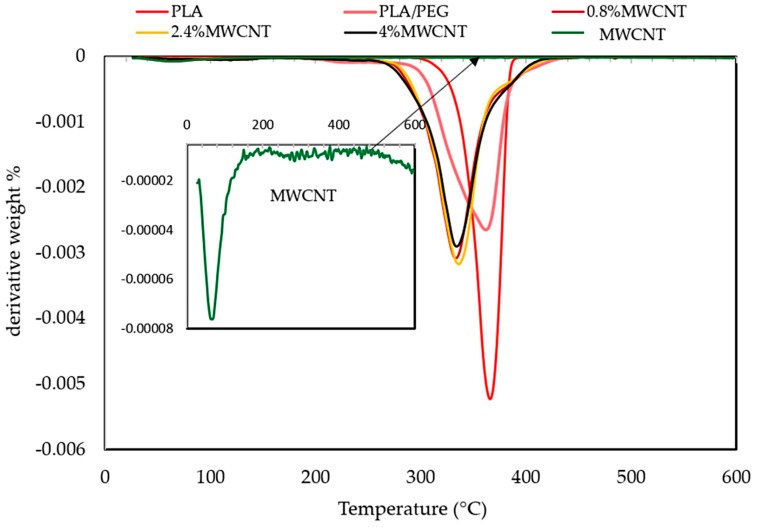
Thermogravimetric (DTG) curves of neat PLA, PLA/PEG blend, MWCNTs powder, and nanocomposites with different MWCNTs loading (0.8 wt.%, 2.4 wt.% and 4 wt.%).

The thermal property investigation of the polymer nanocomposites is necessary for determining the influence of reinforced materials in the polymer matrixes on thermal stability of composites. Additionally, to confirm the presence of process of thermal pyrolysis during composites production, where the EMI shielding material might be subjected to high-temperature conditions during its service life [26]. The thermal stability and degradation properties of the polymeric materials for PLA, PLA/PEG, and MWCNT/PLA/PEG nanocomposites were investigated by (TGA) and (DTG), as shown in the Figure 7 and Figure 8, respectively. Furthermore, the impact of the plasticizer on the thermal stability of the polymeric matrix is studied, where the degradation behavior of polymer molecules is known to be influenced by the presence of the second polymer, because interaction occurred between the polymers. 

Figure 7 showed that the PLA has higher thermal stability than PLA/PEG, because its degradation peak was at 315.6 °C and it was completely decomposed at 387.3 °C. PEG showed peak degradation at 313.5 °C and it was fully degraded at 372.0 °C. The addition of (PEG) to (PLA) lead to molecular weight increase due to the PLA matrix and PEG interaction or molecular chain extension of the PLA matrix itself. Besides that, the presence of PEG was homogeneously dispersed in the PLA polymer, which acts as a barrier sheet [27]. Additionally, Figure 7 shows the influence of the presence of MWCNTs on the thermal stability of the PLA/PEG matrix, where the MWCNTs has excellent thermal stability up to 700 °C and the weight loss was only 0.5% [28].

On the other hand, Figure 7 shows the MWCNT/PLA/PEG nanocomposites’ decomposition between 20 and 600 °C. Accordingly, the weight loss of the composites can be roughly divided into three regions. The first step of weight loss (63–205) °C can be assigned to the loss of adsorbed water in the nanocomposite. The second step of weight loss (205–300) °C can be ascribed to the decomposition of the PEG-PLA polymer mixture. The third loss step (300–388) °C corresponds to the complete breakdown of the polymeric backbone, as well as heavier fragments into still smaller fractions and gaseous by-products. The char residues remaining at (400–600) °C are mainly thermally stable inert materials, like MWCNTs and carbonized polymeric fragments. The thermal stability values of MWCNT/PLA/PEG nanocomposites varied due to the presence of MWCNTs loading when compared to PLA/PEG polymer matrix. Whereas, the decrease in the thermal stability values is a reflection of the high thermal conductivity of nanotubes that may create localized high temperatures in MWCNTs lumps in contrast with the PLA/PEG polymer matrix, where the molecules at the periphery of the lumps may start to degrade earlier. The excellent thermal stability of MWCNTs also result in a reduction to the TGA rate, as shown in Figure 7 and Figure 8 respectively. 

Figure 8 represented the DTG curve of the MWCNT/PLA/PEG nanocomposites, showing that the thermal decomposition mechanism of PLA/PEG mixture is somehow modified in the presence of MWCNTs as a filler. Additionally, the maximum of the first derivative of the TGA curve (DTG) shifted towards higher temperatures with increasing MWCNTs loading. Table 2 records the thermal parameters, such as initial decomposition temperature (T_d,onset_) at 20 °C, the temperature of half decomposition (T_d,50%_), and the temperature of the maximum rate of decomposition (T_d,max_) for different samples at a heating rate of 10 °C/min. obtained from the analysis of TGA curves. The initial decomposition temperature (T_d,onset_) is the temperature at which the loss of weight during heating is just measurable. The temperature of half decomposition (T_d,50%_) is the temperature at which the loss of weight during heating reaches 50% of its final value and the temperature of the maximum rate of decomposition (T_d,max_) is the temperature where the loss of weight reaches to its final value [29]. Furthermore, Table 2 shows the weight loss (%) at decomposition temperature.

### 3.5. Mechanical Properties of the Nanocomposite

**Figure 9:**
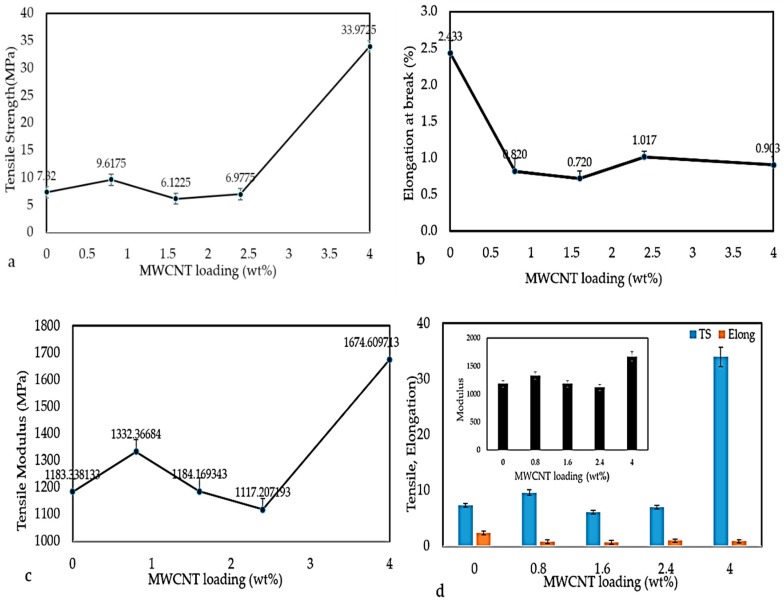
Variation in (**a**) Tensile strength, (**b**) Elongation at break, (**c**) Tensile modulus, and (**d**) Effect of MWCNTs content on a tensile strength (TS), elongation at break (EB), and tensile modulus (TM).

Various factors, like polymer types, filler types, and the degree of dispersion of fillers in the polymer matrix, influence the mechanical properties of the polymer nanocomposites [30]. In present work, the mechanical properties of MWCNT/PLA/PEG nanocomposites containing various MWCNTs contents were examined at room temperature, as is evident in Figure 9a–d. Figure 9a shows the variation in tensile strength (TS) with MWCNTs loading in PLA/PEG nanocomposites. The tensile strength increased with MWCNTs loading from 7.82 MPa (PLA/PEG) matrix to 33.97 MPa of the 4 wt.% MWCNTs. This is due to the addition of stiffer material (MWCNTs) into the polymer matrix, as well the uniform dispersion of MWCNTs resulting in good load transfer from matrix to the MWCNTs, which results in improved mechanical properties at a high loading of MWCNTs filler. Where appropriate, adhesion takes place between the matrix and MWCNTs. 

The elongation at break (EB) of the nanocomposites was recorded at the moment of rupture of the specimen often expressed as a percentage of the original length. It corresponds to the breaking or maximum load. The value of elongation at break (EB), as illustrated in Figure 9b, shows that a PLA/PEG mixture has a higher elongation at break (2.5%) and a reduction with increasing MWCNTs loading. Initially, the elongation at break abruptly decreased after adding the 0.8% MWCNTs loading. Afterward, the trend continued to decrease with a further increase in MWCNTs loading. Increased MWCNTs loading in the matrix resulted in the composites becoming harsh and more solid as the segment mobility of the composites is reduced. This will reduce composites’ resilience and toughness and lead to lower resistance to breaking.

The tensile modulus (TM) is considered to be a common method for the measurement of the material’s stiffness. It is also a quantity used in characterizing materials [31]. The higher values of tensile modulus indicate higher material’s stiffness; thus, more stress will be needed to produce a given amount of strain. Figure 9c shows that the tensile modulus of PLA/PEG matrix had a high tensile modulus (1183.33 MPa). The addition of 0.8 mass % of MWCNTs to the matrix leads to a slight increase in the tensile modulus and hence the stiffness. Additionally, the tensile modulus when adding 1.6 wt.% and 2.4 wt.% of MWCNTs gradually decreased. This might result from the fact that interaction between nanoparticles can take place after the rotary relaxation via bridging by polymer chains or direct contacts. This signifies that the material is less in terms of tensile modulus when compared to the PLA/PEG matrix. However, at higher concentrations of MWCNTs (4 wt.%), the close contact between MWCNTs clusters can give rise to a rigid filler network, which increases the tensile modulus. Figure 9d illustrates the effect of MWCNTs loading on all parameter TS, EB, and TM.

### 3.6. Dielectric Properties of MWCNT/PLA/PEG Nanocomposites

**Figure 10:**
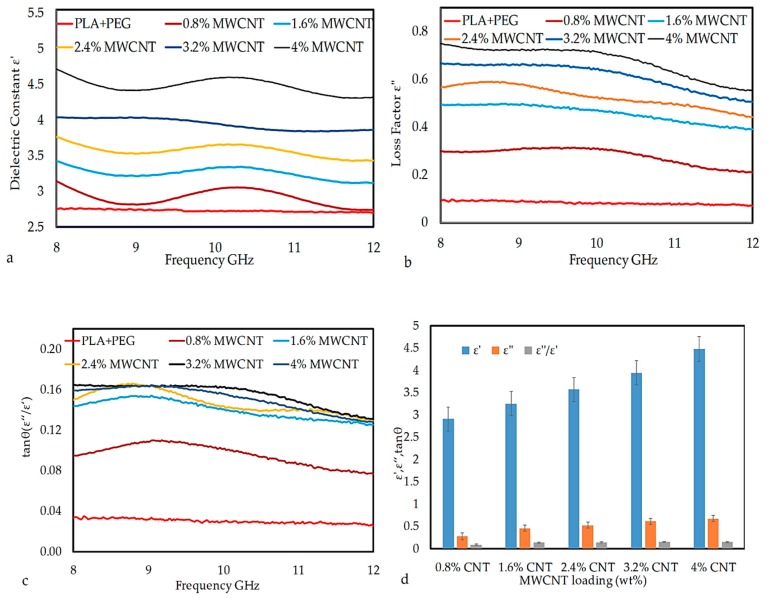
The plot of (**a**) ε′, (**b**) ε″, (**c**) tan δ vs. frequency, and (**d**) comparison of ε′, ε″, and tan δ as a function of MWCNTs nanoparticle loading at MWCNT/PLA/PEG nanocomposites.

MWCNTs nanoparticles always contain lattice defects, like vacancies, interstitial bonding, and CO or OH attachments [32], which act as active centers for the interaction of polymeric chains on the surface of MWCNTs and enhance real part of permittivity. Similarly, the imaginary part of permittivity in MWCNT/polymer composites increases with an increasing MWCNTs content due to high conduction current. Figure 10a,b show frequency-dependent spectra of the real and imaginary part of the permittivity of composites containing varying amounts of MWCNTs. It is observed that the real and imaginary part of permittivity both increase with increasing MWCNTs content and decrease with increasing frequency from 8 to 12 GHz. At lower loadings of MWCNTs, frequency has little effect on both the real and imaginary parts of the permittivity, but the significant decrease is observed at higher loadings, e.g., in sample 3.2% MWCNTs and 4% MWCNTs. The effect of frequency on permittivity is directly related to polarization, i.e., as the frequency of the field is raised, the periodic reversal of electric field occurs so quickly that there is no excess ion diffusion in the field direction [33]. 

Hence, polarization due to charge accumulation decreases, which leads to a decrease in the permittivity value with increasing frequency [34]. Such an increase in both real and imaginary parts of permittivity is due to an increase in conductivity and dipole moment of MWCNT/PLA/PEG nanocomposites. It is also interesting to note that, at higher MWCNTs loadings, the real and imaginary part of permittivity shows a fluctuation in the measured frequency range, like a broad peak can be seen between 9 and 11 GHz. This result suggests the existence of a resonance phenomenon, which is expected in the case of highly conductive composites as the skin effect becomes significant [35]. The loss tangent (tan δ) that is commonly used to describe dielectric losses, [36] is calculated by using Equation (1) and is plotted in Figure 10c.

(1) $$\mathrm{Tan}\;\delta \;=\;\;\;\frac{\varepsilon^{\prime \prime }}{\varepsilon^{\prime }}$$

Figure 10c shows the values of tan δ versus frequency 8–12 GHz of MWCNT/PLA/PEG nanocomposites as a function of MWCNTs nanoparticle loading. The tan δ values of MWCNT/PLA/PEG nanocomposites increased with an increase in MWCNTs loading and vice versa for frequency. The increase in tan δ value of PLA/PEG upon the incorporation of MWCNTs nanoparticle can be attributed to the phase transition of material, i.e., conversion from an insulator to conducting material. Figure 10d illustrates the column chart of the variations of the ε’, ε’’, and tan δ of the MWCNT/PLA/PEG nanocomposites. In the column chart, a function of MWCNTs nanoparticle loading has been organized along the horizontal axis and ε’, ε’’, and tan δ values along the vertical axis. It can be seen that the permittivity of nanocomposites is very sensitive to MWCNTs loading. The ε’, ε’’, and tan δ display an increasing trend with an increase in filler loading over the X-band frequency range. This significant improvement in permittivity is a result of the increase in the dipole moment and conductivity of MWCNT/PLA/PEG nanocomposites due to the addition of MWCNTs.

### 3.7. EMI Shielding Mechanism of Nanocomposites

#### 3.7.1. Shielding Effectiveness

The EMI SE of a material is defined as the attenuation of propagating electromagnetic waves that are produced by the shielding materials. The total shielding effectiveness (SE total) can be expressed and is described as the sum of the contribution due to absorption (SE_A_), reflection (SE_R_), and multiple reflections (SE_M_), as follows [30]:

SE total (dB) = SE_A_ + SE_R_ + SE_M_ (2)

On the other hand, the SE_M_ is a correction term whose value might be positive, negative, or zero. For this reason, the effect of multiple reflections between both interfaces of the material is negligible [37]; therefore, the total shielding effectiveness (SE total) will be expressed as:

SE total (dB) = SE_A_ + SE_R_ (3)

Therefore, the experimental absorption loss (SE_A_) and the reflection loss (SE_R_) can be written as:

SE_A_= −10 log[Tr/(1 − Re)] (4)

SE_R_= −10 log (1 − Re) (5)

where, reflection (Re) and transmission (Tr) coefficients are represented by the S_11_ and S_12,_ and they are the scattering parameters of the two-port vector network analyzer (VNA) system, respectively.

**Figure 11:**
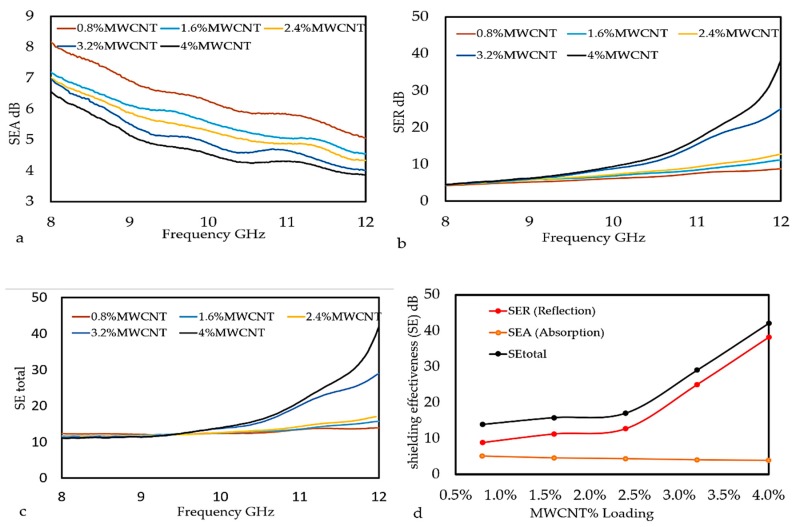
Variation of Shielding Effectiveness, (**a**) sum of the contribution due to absorption (SEA), (**b**) reflection (SER), and (**c**) shielding effectiveness (SE total) as a function of frequency measured in X-band, and (**d**) Comparison of SE total, SER, and SEA as a function of MWCNTs nanoparticle loading at MWCNT/PLA/PEG nanocomposites.

The impact of MWCNTs filler and X-band frequency range to the EMI SE of MWCNT/PLA/PEG nanocomposite was studied by incorporating different percentages of MWCNTs into the PLA/PEG polymer matrix. Figure 11a–d presents the EMI SE measurement at an 8–12 GHz frequency range. Figure 11a presents the inverse proportional of SEA values to MWCNTs loading and frequency range, which results from the increase in conductivity along with the capacitive coupling effects [38]. A directly proportional relation of SER values to both MWCNTs content and used frequency is clearly shown in Figure 11b. This might be due to the shield impedance and skin depth increase with frequency [39]. Equation (3) was used to calculate the SE total values. Figure 11c displays the high SE total values affined to the high filler% and frequency, which results in a high EMI total of MWCNT/PLA/PEG nanocomposites. Where, the lowest EMI SE value of 13.879 dB was recorded at 0.8 wt.% of MWCNTs loadings, while a 42.078 dB was recorded at 4 wt.% and 12 GHz. The overlapping of the EMI SE curves appears until (<9.5 GHz) frequency range and then separated afterward (>9.5 GHz). Figure 11d shows the comparison EMI-shielding performance as a function of MWCNTs nanoparticle loading at MWCNT/PLA/PEG nanocomposites. Whereas, the high value of EMI SE total that was obtained at a high loading of MWCNTs could be attributed to the fine dispersion and distribution of conducting MWCNTs in the PLA/PEG polymer matrix, thereby forming the coordinated conducting network. The results show that the change at EMI SE leads to shifting materials behavior, which is the manifestation of change of intrinsic properties of the nanocomposites, i.e., the nanocomposites conversion from a state to another status with different properties [40]. For EMI shielding efficiency, the electrical behaviors of the material are very important, since they are responsible for interacting with the electromagnetic wave, where the total EMI SE is affected by the number of mobile charge carriers provided by the filler network in the composites and mesh size [41]. However, the effective utilization of MWCNTs for fabricating nanocomposites depends strongly on the homogeneous dispersion of MWCNTs throughout the PLA/PEG polymer matrix without destroying their integrity. The higher values of the SE results of the MWCNT/PLA/PEG nanocomposites than the maximum value of EMI SE of the shielding that is required for practical applications, which is usually rated around 20 dB.

Table 3 shows the experimental results of the overall EMI SE (SEA, SER, and SE total) for all the samples at different percentages of the MWCNTs at 12 GHz. The results show that the SEA is much lower when compared to SER results, while the SE total is higher than the SER and SEA values.

The EMI SE total results of the MWCNT/PLA/PEG nanocomposites in the current work were compared with other prepared nanomaterials that were based on various polymers at different loading percentages, different sample thicknesses at X-band, and then tabulated in Table 4. the results show the EMI SE herein crossed the limit value of EMI SE of the shielding that is required for practical application is usually considered to be (~20 dB), which suggests that these nanocomposites are promising candidates for manufacturing a material shielding. 

#### 3.7.2. The Conductivity of MWCNT/PLA/PEG Nanocomposites

The scientists and researchers have been interested in using the MWCNTs composite as absorber materials and electrical conductors due to interesting electromagnetic characteristics, including good microwave absorption, high electrical, and conductivity [51]. This is because MWCNTs contain relatively large amounts of carbon, which can increase the radiation absorption performance and dielectric properties [52]. Therefore, the connectivity between the filler particles is a paramount demand for high conductivity and also for the enhancement of EMI SE. High conductivity will enhance shielding effectiveness by interconnecting network particles. The conductivity of the nanocomposite has been calculated by using the loss factor of dielectric properties that were obtained at the X-band via Equation (6), below [53]:

(6) $$\sigma_{MWCNT}=2\pi f\varepsilon_{O}\varepsilon^{\prime \prime }$$

where *σ* is the conductivity (S/m), *f* is the frequency (GHz), $\varepsilon_{o}$ is the dielectric in free space, and *ε’’ is* the imaginary part of the dielectric properties. 

**Figure 12:**
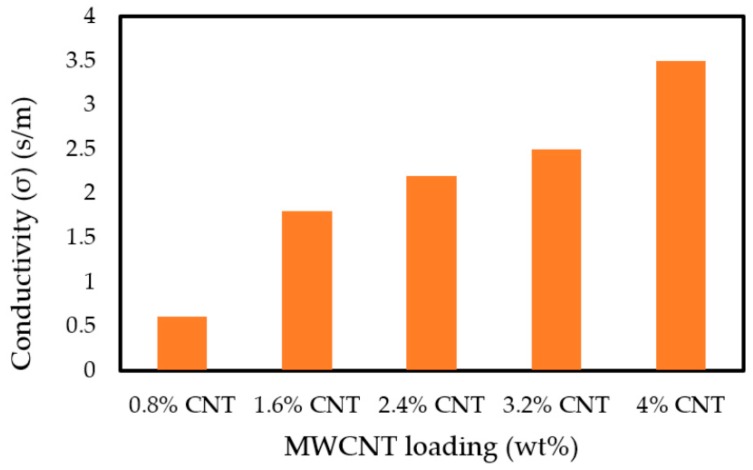
The conductivity of the MWCNT/PLA/PEG nanocomposites specimens as a function of MWCNTs content.

The MWCNTs dispersed in a polymer matrix can enhance the conductivity of the conductive polymer nanocomposite. Figure 12 shows the influences of the MWCNTs content on the conductivity. The conductivity of the MWCNT/PLA/PEG nanocomposites increased as the content of MWCNTs increased. In addition, the conductivity of the MWCNT/PLA/PEG nanocomposites increased as the frequency increased because conductivity is proportional to the frequency; refer to Equation (6). For example, the 0.8 wt.% sample had average values of conductivity with 0.61 S/m in the frequency region. The MWCNT/PLA/PEG nanocomposites became conductive with high dielectric properties when the weight percentages of MWCNTs gradually increased.

#### 3.7.3. Power Balance

The power data collected from the EMI shielding characterization set-up were analyzed to find the effect of absorption and reflection of the overall shielding of MWCNT/PLA/PEG on the nanocomposites. The transmission power (Tr) and the reflection power (Re) were calculated by the equations:

Tr = |S_21_|^2^, Re = |S_11_|^2^ (7)

The absorbed power (A) and absorption efficiency (AE%) indicate the attenuation contribution of electromagnetic absorption when the waves travel into the materials, both of which can be calculated using;

A = I − (Tr + Re)(8)

AE (%) = (A/1 − Re) × 100(9)

The measured reflected power Re is not only the power that has been reflected from the external surface, but it also includes the positive contribution of internal surface reflection and negative contribution of multiple-reflection as well [54]. 

**Figure 13:**
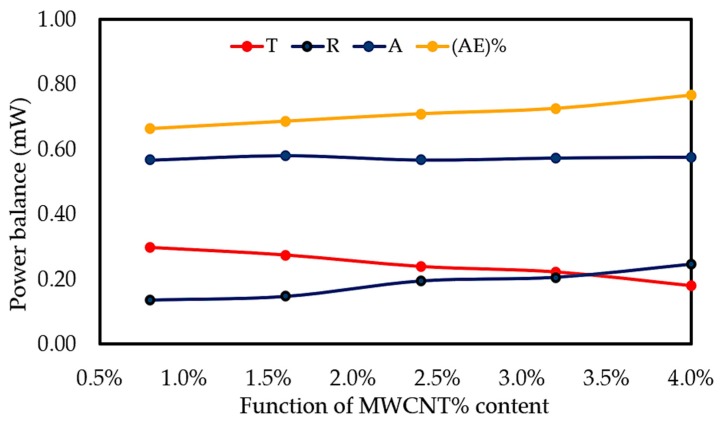
Power balance in the X-band frequency range for 3 mm plates made of MWCNT/PLA/PEG nanocomposites as a function of MWCNTs filler content.

Figure 13 represents the amount of the transmitted (Tr), reflected (Re), and absorbed power (Ab) of MWCNT/PLA/PEG nanocomposites as a function of MWCNTs concentration at 3 mm thicknesses. In addition, the figure includes the Absorption efficiency (AE)%, where the total (AE)% for all of the samples were found to decrease with an increasing frequency, while the maximum (AE)% was observed to reach the highest values at higher MWCNTs loading, demonstrating higher efficiency in the nanocomposites. The energy that is attenuated by absorption is generally converted into heat [55]. The lower amount of power blocked by absorption is due to the lower power transmitted into the sample as a result of the better reflection. The contribution of absorption to the overall shielding should be based on the ability of the material to attenuate the power that has not been reflected. Generally, one portion of waves have been reflected and the other waves travel into the material when the electromagnetic waves reach the surface of EMI shielding material, as shown in Figure 3c. The amplitude of the reflected waves and transmitted waves through a material depends on the impedance of the material and impedance of the medium in which incident electromagnetic waves travel. Therefore, the MWCNT/PLA/PEG nanocomposites with higher MWCNTs loading exhibit much lower impedance, thus exhibiting greater impedance mismatch and higher reflection. Table 5 shows the results of power balance of MWCNT/PLA/PEG nanocomposites as a function of MWCNTs content. 

**Figure 14:**
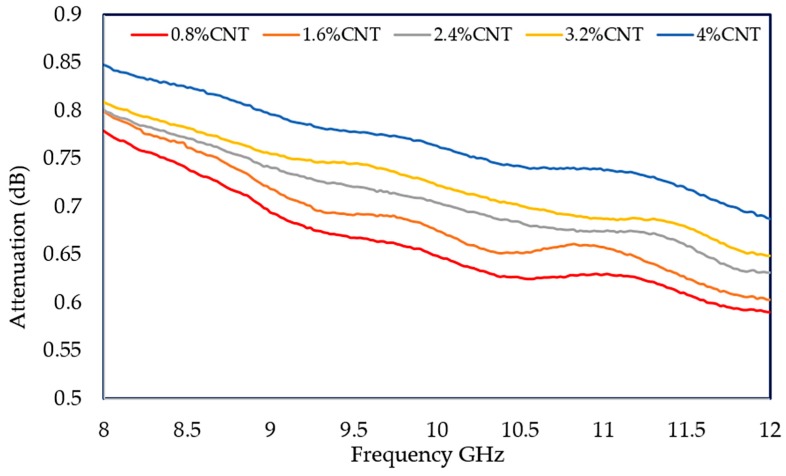
Electromagnetic waves attenuation in the MWCNT/PLA/PEG nanocomposites at X-band frequency.

The mechanism for dielectric properties and the microwave response is associated with the microwave attenuation capacity. The attenuation properties mainly originate from the electric loss of MWCNTs by the motion of conducting electrons [56]. Therefore, the attenuation of electromagnetic waves of MWCNT/PLA/PEG nanocomposites has been experimentally investigated with a thickness of 3 mm at 8–12 GHz, as revealed in Figure 14. The attenuation values of the nanocomposites increase with an increase in MWCNTs loading and reach the maximum values of 0.85 at the higher percentages for filler and low frequency. Additionally, the attenuation values of the MWCNT/PLA/PEG nanocomposites are in the range of (0.77–0.85) dB.

## 4. Conclusions

The MWCNT/PLA/PEG nanocomposites were successfully fabricated for EMI shielding applications and their properties were investigated. FE-SEM microphotographs showed that the MWCNTs are well-dispersed in the PLA/PEG matrix. The thermal stability test confirmed that the incorporation of MWCNTs reduce the thermal stability values of the MWCNT/PLA/PEG nanocomposites when compared to the PLA/PEG polymer matrix. The effect of multi-walled carbon nanotubes on mechanical properties TS, EB, and TM on the composite is noticeable. This fact correlates to the increase of both tensile strength and tensile modulus, while the elongation at break decreased for nanocomposite as compared to the PLA/PEG polymer matrix. Dielectric properties, conductivity, and the EMI SE of the nanocomposites were measured at 8–12 GHz frequency range, where it can be observed that the MWCNTs improved the dielectric properties and conductivity of MWCNT/PLA/PEG nanocomposites. Furthermore, the MWCNTs were able to achieve the shielding levels that were required for various industrial applications without compromising the physical properties of the polymer matrix. The results show that the MWCNT/PLA/PEG nanocomposites with 4 wt.% of MWCNTs have an excellent EMI shielding ability of 42.078 dB and the product is eligible for meeting the commercial application of EM shielding requirements, especially at the X-band range.

## Figures and Tables

**Table 1:** The compositions of the nanocomposites.

Sample	Weight % of PLA	Weight % of PEG	Weight % of MWCNTs	Mass (gm) MWCNT/PLA/PEG
MWCNT/PLA/PEG	90.00	10	0	25 gm
	89.28	9.92	0.8	
	88.56	9.84	1.6	
	87.84	9.76	2.4	
	87.12	9.68	3.2	
	86.4	9.60	4	

**Table 2:** Thermal properties and weight loss parameters of the PLA, MWCNTs powder, PLA/PEG blend, and MWCNT/PLA/PEG nanocomposites.

Sample	Tonset °C	*T*_50%_ °C	*T*_*d−max*_ °C	Weight Loss %
PLA	315.66	365	387.33	98.7
PLA/PEG	313.5	335	372.0	92.4
MWCNT	62.66	227	241.8	5
0.8% MWCNT	277.33	334.4	371.8	95.3
2.4% MWCNT	275.37	335.6	373.6	95.5
4% MWCNT	273.5	335.6	377.3	96.2

**Table 3:** Results of shielding effectiveness of MWCNT/PLA/PEG for different MWCNTs loading at 12 GHz.

Filler [wt.%]	SEA	SER	SEtotal
0.8	5.053	8.826	13.879
1.6	4.551	11.226	15.777
2.4	4.333	12.684	17.018
3.2	4.013	25.016	29.029
4%	3.873	38.206	42.078

**Table 4:** Comparative of EMI shielding performance of present work to different polymer composites [42].

Composites	Filler Content	Thickness (mm)	EMI SE (dB) at 8–12 GHz	References
MWCNT/Polypropylene	7.5 vol %	1.0	~34	[43]
SWCNT/Epoxy	15 wt.%	2	25	[44]
MWNCT/Polyacrylate	2 wt.%	1.5	~4	[45]
MWNCT/Polystyrene	7 wt.%	1	26	[46]
SWCNT/Polyaniline	20 wt.%	2.4	19	[47]
MWNCT/Polyurethane	10 wt.%	2.5	~41.6	[48]
MWNCT/Poly(trimethylene terephthalate)	7.5 wt.%	2	~23	[49]
MWNCT/Epoxy	20.4 wt.%	0.35	~19	[50]
MWCNTs/MnZn Ferrites/Epoxy	4.0 vol %	2.0	17	[42]
MWCNT/PLA/PEG	0.8%	3	42.078	Present work

**Table 5:** The results of the power balance of MWCNT/PLA/PEG nanocomposites as a function of MWCNTs content.

MWCNTs %	T	R	A	(AE) %
0.8	0.297	0.136	0.567	0.662
1.6	0.274	0.147	0.579	0.685
2.4	0.239	0.194	0.567	0.709
3.2	0.222	0.206	0.573	0.725
4	0.179	0.246	0.575	0.766
